# Unexpected Internal Mammary Vein Cannulation by an Implantable Central Venous Catheter: A Rare Complication

**DOI:** 10.7759/cureus.105615

**Published:** 2026-03-21

**Authors:** Luke Guy, Gagan Japra, Herjot Cheema, Sanjay Munireddy

**Affiliations:** 1 Surgery, Phoebe Putney Memorial Hospital, Albany, USA

**Keywords:** central venous catheter (cvc), cvc malposition management, implantable cvc port, misplacement of cvc, surgical case reports

## Abstract

Central venous catheters (CVCs) play an integral role in the management of complex patients. Approximately 2% of CVC placements are improperly placed. One exceedingly rare form of malposition is placement within the internal mammary vein. Misplacement within this vein is difficult to diagnose due to its course overlying the right cardiac border on routine chest imaging, as well as being typically subclinical in presentation.

We present a case of a 77-year-old female, recently diagnosed with ovarian cancer, who was referred to the surgical oncology service for placement of an implantable CVC for neoadjuvant chemotherapy. Initial implantable CVC placement was conducted via left subclavian approach, which was confirmed by routine intra-operative fluoroscopy to be overlying the right cardiac border, and presumably within the superior vena cava. Intra-operatively, the catheter was withdrawn with dark venous blood, flushed without difficulty, and the patient experienced no hemodynamic instability. The patient tolerated chemotherapy well with no complications immediately following implantable CVC placement, but there was difficulty in withdrawing blood from the port site by floor nursing on post-operative day one. Follow-up lateral view fluoroscopy discovered the catheter to be misplaced within the right internal mammary vein. This was an initial misplacement that was missed due to the anatomic course of the internal mammary vein and how it can mimic correct placement on routine intra-operative and post-operative imaging. Three weeks later, the patient underwent elective repositioning of the catheter into the superior vena cava (SVC) at the right atrial junction under fluoroscopy with additional use of Omnipaque contrast solution to ensure accurate placement. She was discharged home the same day in stable condition. This case demonstrates the importance of multiview imaging to confirm proper CVC placement. Because the right internal mammary vein overlies the right cardiac silhouette on an anteroposterior chest X-ray, the patient’s implantable port appeared to be correctly placed in the SVC. Imaging modalities involving fluoroscopy or chest CT can be alternatives to confirming successful access to the SVC.

## Introduction

Central venous catheters (CVCs) play an integral role in the treatment of disease among complex patients. One type of CVC, commonly referred to as a port-a-cath, is a type of tunneled catheter meant for long-term use. The implantable “port” resides under the skin and utilizes the body's natural barrier to help reduce infection. While malpositioning of the CVC is considered rare, some reports have cited an incidence as high as 2%. A study from Muhm et al. reported ~2% malposition rate [1]. While ultrasound-guided techniques have improved successful catheter placement in the internal jugular vein (IJV), studies have failed to show significant improvements in the malpositioning of the catheter tip. This is likely because ultrasound does not allow the operator to visualize the entire aspect of the CVC and can be inadvertently misplaced within the venous vasculature distally, and will not be found unless post-procedure imaging is obtained. In a study by Keum et al., no significant improvement in malpositioning with ultrasound guidance was found [2].

Common complications of CVC placement include malposition, arterial puncture, pneumothorax, hematoma, and infection. Malposition can occur due to a variety of factors. Some studies have shown that the operator’s orientation of the inserting bevel, in this case caudally for a subclavian approach, may correlate with higher rates of appropriate catheter placement [3]. Other causes of malpositioning include factors related to tip migration due to the patient’s body habitus and normal variations in venous anatomy. This complication can present with life-threatening dysrhythmias, chest pain, inadequate blood return from the venous access point, and pain during injections through the catheter.

Malpositioning into branches of the brachiocephalic vein, such as the jugular or contralateral brachiocephalic, is common; however, these are typically detected upon routine post-operative chest X-ray. Malposition within the right internal mammary vein presents unique diagnostic difficulties, due to its course overlying the right heart border. This has the potential to be missed on a routine chest X-ray post-operatively, as was the case with this procedure.

## Case presentation

**Figure 1:**
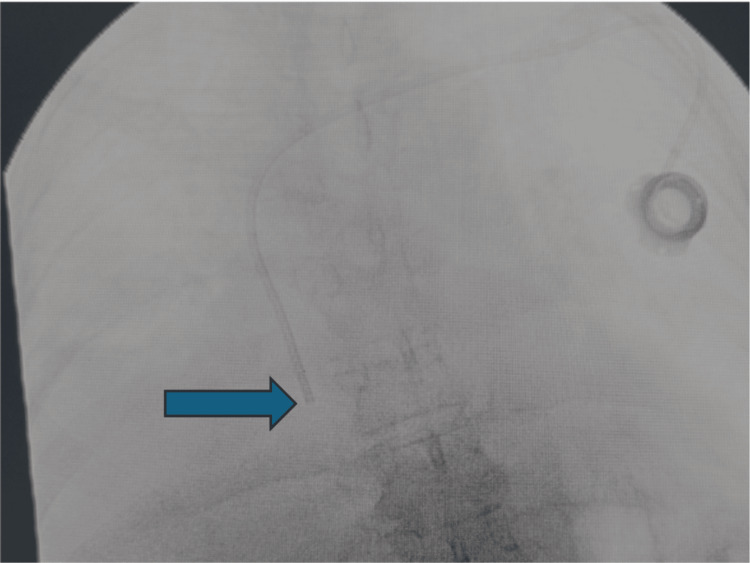
Intra-operative fluoroscopy from initial placement. The catheter tip is seen overlying the right cardiac silhouette just above the cavo-atrial junction. The blue arrow shows the catheter tip position.

We present a case of a 77-year-old female with recently diagnosed metastatic ovarian cancer who was referred to the surgical oncology service for implantable CVC placement to begin receiving neoadjuvant chemotherapy (NAC). The initial approach for placement was performed via the left subclavian vein. The implantable CVC catheter was placed under fluoroscopic guidance, and the tip of the catheter was readjusted over the right heart border and was presumed to be within the superior vena cava (SVC) right atrial junction. This was ultimately determined not to be the case, as will be discussed later. Figure 1 shows the initial intra-operative fluoroscopic image demonstrating catheter placement during the procedure.

As shown, the catheter tip overlies the right heart border near where a correctly positioned CVC should terminate. As a result, it was initially thought to be positioned appropriately, but that was not the case, and the initial images were ultimately misinterpreted by the surgical team. Intra-operatively, the port was accessed, aspirated, and flushed without difficulty. The port was positioned within a subcutaneous skin flap, and the skin was closed. The patient tolerated the procedure well and was brought to post-anesthesia care unit (PACU) in a stable condition. Post-operative chest X-ray was performed and revealed no evidence of pneumothorax and re-demonstrated the tip of the catheter near the SVC-right atrial junction. This was determined to be a CVC malposition, a rare complication of CVC placement. An overview of complications associated with CVC placement and their respective rates is presented in Table 1. This is adapted from a meta-analysis conducted by Teja et al. [4].

**Table 1:** Rates of complications associated with CVC placement. CVC: central venous catheter

Complication	Events per 1000 catheters placed (95% credible interval)
Placement failure	20.4 (10.9-34.4)
Arterial cannulation	2.8 (0.1-10)
Arterial puncture	16.2 (11.5-22)
Pneumothorax	4.4 (2.7-6.5)

**Figure 2:**
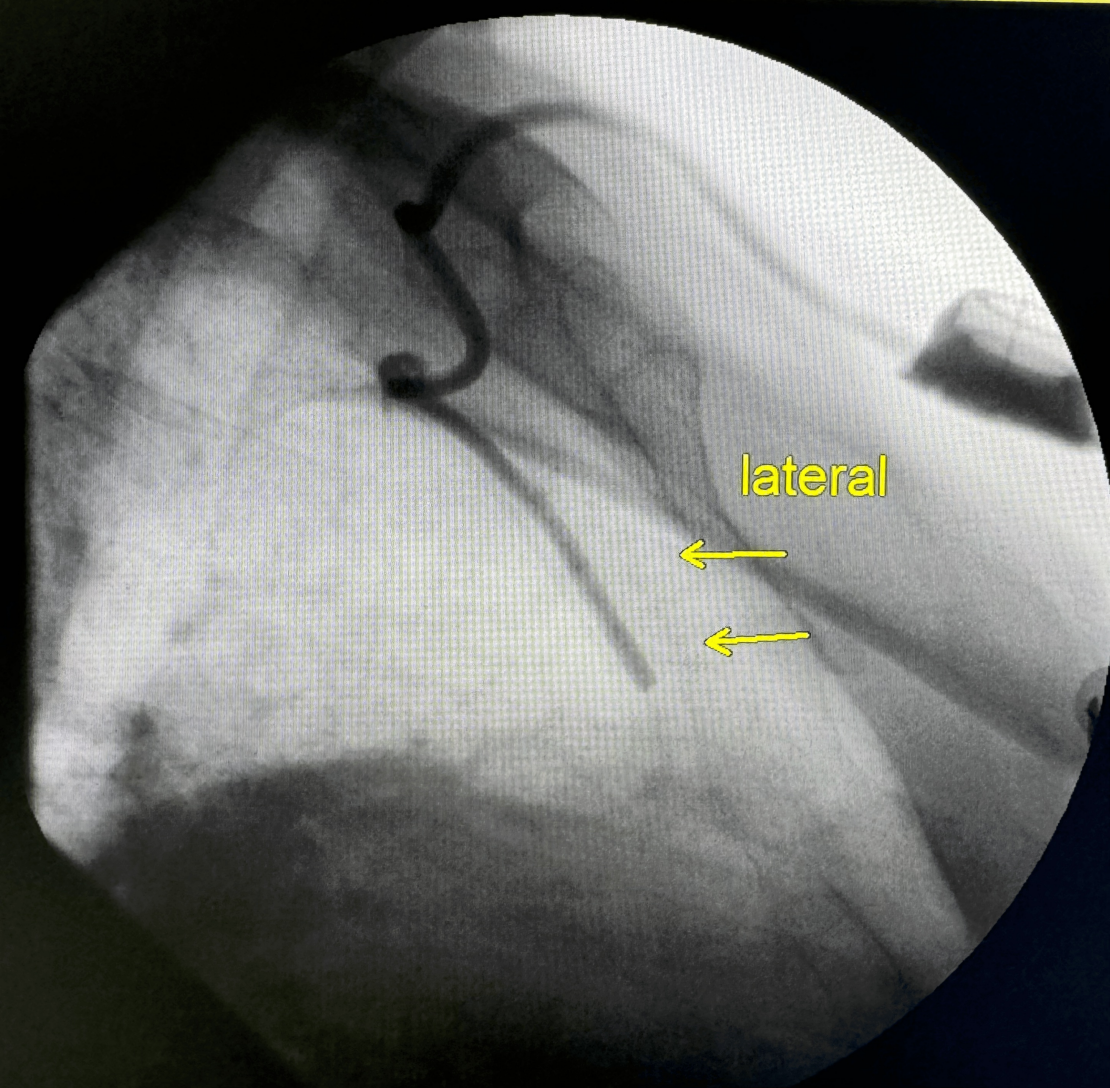
Lateral view fluoroscopy obtained after CVC would not draw blood on the floor. CVC: central venous catheter

On post-operative day (POD) one, the patient remained stable and was scheduled to undergo initial chemotherapy. Nursing staff had difficulty drawing blood from the implantable CVC hub for morning labs. The decision was made to repeat both AP and lateral view fluoroscopy. The lateral view revealed that the catheter was coursing along the anterior thoracic wall, presumably within the right internal mammary vein (Figure 2). 

A multidisciplinary discussion was held, and the decision was made to leave the CVC malpositioned so that the patient had no further delays toward beginning neoadjuvant chemotherapy, with the potential for elective repositioning of the port in the future, so that the patient may receive lab draws from their port or if complications arise.

On POD two, the patient began inpatient NAC. She tolerated chemotherapy well without significant complications and was discharged 11 days after beginning NAC. Approximately three weeks after initial CVC implantation, the patient was brought back to the operating room for catheter repositioning to avoid multiple venipunctures. There were no interval complications related to the initial malposition.

**Figure 3:**
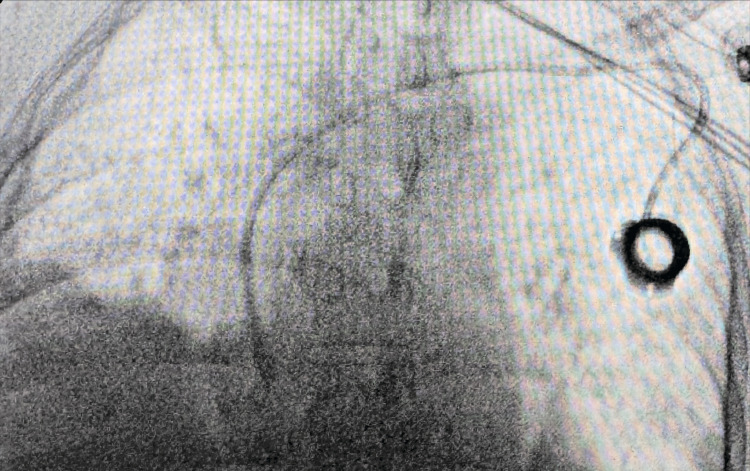
Intra-operative fluoroscopy after the replacement of the CVC within the right atrium. CVC: central venous catheter

The previous incision below the left clavicle was reopened and enlarged. Dissection was carried down until the catheter was visualized. Under fluoroscopy, the catheter was gently retracted until it was within the left innominate vein. Multiple attempts were made to advance the catheter into the right atrium; however, it continued to advance to the right internal mammary vein. On the third attempt, the catheter was felt to advance into the right atrium. Intra-operative fluoroscopy images are located in Figure 3.

**Figure 4:**
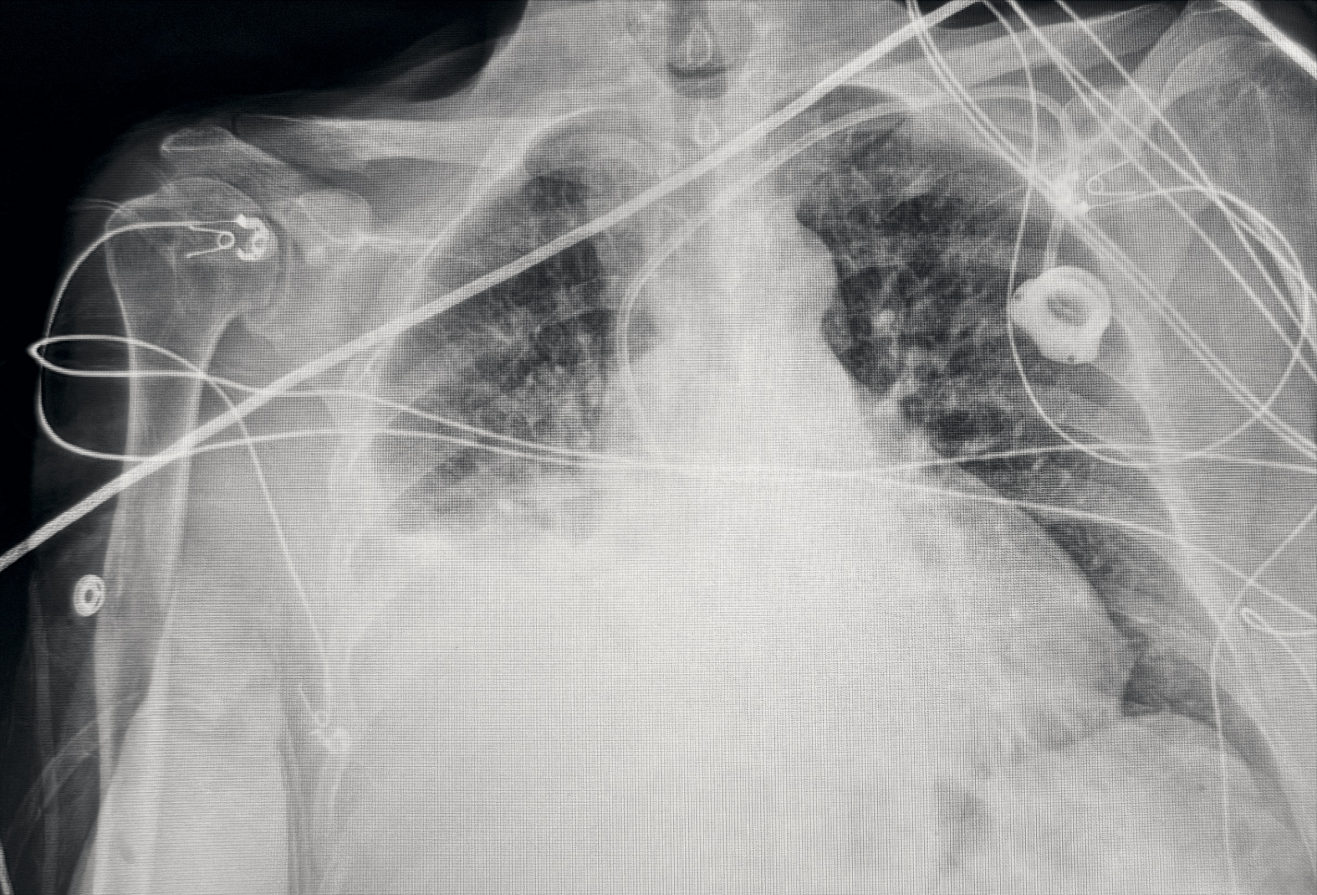
Final post-operative chest X-ray after replacement.

To confirm accurate placement, Omnipaque contrast was injected into the port through a Huber needle to definitively confirm placement with intra-operative fluoroscopy. Intra-operatively, contrast and fluoroscopy were used in both AP and lateral orientation to definitively confirm that the tip was residing within the SVC. Contrast was seen flowing within the central venous vasculature, and not within the internal mammary vein. The port was aspirated and flushed without difficulty, and the incision was closed. The patient tolerated the procedure well and was brought to PACU in stable condition. A post-operative chest X-ray was obtained and is shown in Figure 4. The patient was discharged without complication. This patient’s port remained functional and continued to receive treatments as well as laboratory draws through the repositioned port. The patient unfortunately passed away from their malignancy one and a half years following their initial treatment.

## Discussion

This case demonstrates a rare complication of CVC placement. Cannulation of the left or right internal mammary vein is an exceedingly rare complication of CVC insertion, and in this literature review, the majority of cases observed occurred on the left side from a left-sided IJV or subclavian vein (SCV) approach (Munta et al., Goodin et al., and McLean II et al.), suggesting that anatomic factors are at play [5-7]. The left brachiocephalic vein traverses the mediastinum horizontally, giving rise to multiple anterior thoracic tributaries, including the internal mammary vein (IMV), which may predispose a catheter directed anteriorly to enter the vessel. The right brachiocephalic vein follows a shorter and more vertical course into the SVC, potentially making right IMV cannulation less likely.

Accidental cannulation of the left internal mammary vein is easily identified on post-procedure or intra-operative chest X-ray, but cannulation of the right IMV is more subtle, as the catheter tip may still overlie the right cardiac silhouette on imaging. There are, however, subtle differences that can be seen between the initial malpositioned imaging and the corrected placement confirmed with Omnipaque. In the first image, the angle at which the catheter passes from the right brachiocephalic into the right IMV is very sharp, almost 90°, whereas in the correctly placed image, a slightly less abrupt angle is seen as the catheter transitions from the right brachiocephalic into the superior vena cava. This subtle finding should prompt suspicion for displacement within the anterior thoracic venous system and may be further evaluated using lateral or oblique fluoroscopic views to assess whether the CVC courses along the anterior thoracic wall.

While malposition within the right IMV has been documented before, as reported by Sakan et al. and Tristão et al., although sparingly, even less documented is a CVC malpositioned within the right IMV from a left subclavian approach [8,9]. While the technical and anatomical aspects of malposition within this vein are the same, removal of an implantable CVC is not as straightforward as removal of a generic central line. The decision to proceed with chemotherapy despite the malposition was made following a multidisciplinary joint discussion where it was determined that it would be safe for the patient to have her chemotherapy administered with the CVC positioned within the IMV. In her case, the catheter remained fully intravascular with unobstructed flow, and imaging showed no evidence of extravasation or vascular injury. This decision was based on a growing body of literature regarding utilization of the IMV for central venous access among patients who had lost their central IV access due to chronic need for central access, as reported by Alomari et al. [10]. This study’s findings suggest that the IMV can function as a viable conduit for catheter-based infusion when central access is confirmed. Although the present case involved unintended cannulation, this evidence supports the decision to temporarily use the catheter while closely monitoring for complications. The patient was able to tolerate their initial chemotherapy well for around three and a half weeks and then went on to have the catheter replaced within the SVC at a later date, so that the patient may have the benefit of not requiring multiple peripheral IV accesses for laboratory tests while undergoing chemotherapy.

This case underscores the diagnostic challenge of right IMV cannulation, which can mimic correct positioning on anterior-posterior chest imaging. Multiview fluoroscopy or cross-sectional imaging should be employed whenever catheter function is compromised despite apparently correct positioning. In select cases, a malpositioned but functionally patent catheter may be temporarily used for low-risk infusions under close monitoring, though definitive correction should be pursued to prevent long-term complications. Awareness of this rare but clinically significant pitfall is essential for surgeons and interventionalists performing CVC placements.

## Conclusions

Overall, this case demonstrates a rare CVC malpositioning within the right internal mammary vein. While single-view imaging remains routine for these procedures, this study may be of minimal utility in this specific malposition because of the course of the right IMV overlying the right heart border. If malposition within the right IMV is suspected, either due to the 90° course of the CVC or due to difficulty withdrawing blood, clinicians should obtain a lateral view imaging to determine the exact course of the CVC. If malposition is diagnosed, the catheter should generally be replaced within the SVC promptly. However, if the exchange of said catheter could lead to delays in the patient's care that would have an impact on their clinical outcome, the internal mammary veins can be utilized in an inpatient setting, where complications can be closely monitored in select cases. Further studies are warranted to investigate the long-term outcomes of the utilization of the IMV for central venous access before it can be considered a safe alternative route for central line placement.
